# Effects of transcranial direct current stimulation (tDCS) on posture, movement planning, and execution during standing voluntary reach following stroke

**DOI:** 10.1186/s12984-020-00799-8

**Published:** 2021-01-07

**Authors:** Chieh-ling Yang, Alon Gad, Robert A. Creath, Laurence Magder, Mark W. Rogers, Sandy McCombe Waller

**Affiliations:** 1 Department of Physical Therapy and Rehabilitation Science, University of Maryland School of Medicine, 100 Penn Street, Baltimore, MD 21201 USA; 2 Department of Epidemiology and Public Health, University of Maryland School of Medicine, Baltimore, MD 21201 USA; 3 Present Address: Rehabilitation Research Program, Vancouver Coastal Health Research Institute, 4255 Laurel Street, Vancouver, BC V5Z2G9 Canada; 4 Department of Physical Therapy, University of British Columbia, Vancouver, BC V6T1Z3 Canada; 5 Lewis Human Performance Lab, Department of Exercise Science, Lebanon Valley College, Annville, PA 17003 USA; 6 Division of Health, Business, Technology and Science, Frederick Community College, 7932 Oppossumtown Pike, Frederick, MD 21702 USA

**Keywords:** Transcranial direct current stimulation, startReact, Motor preparation, Postural control, Reach, Stroke

## Abstract

**Background:**

Impaired movement preparation of both anticipatory postural adjustments and goal directed movement as shown by a marked reduction in the incidence of StartReact responses during a standing reaching task was reported in individuals with stroke. We tested how transcranial direct current stimulation (tDCS) applied over the region of premotor areas (PMAs) and primary motor area (M1) affect movement planning and preparation of a standing reaching task in individuals with stroke.

**Methods:**

Each subject performed two sessions of tDCS over the lesioned hemisphere on two different days: cathodal tDCS over PMAs and anodal tDCS over M1. Movement planning and preparation of anticipatory postural adjustment-reach sequence was examined by startReact responses elicited by a loud acoustic stimulus of 123 dB. Kinetic, kinematic, and electromyography data were recorded to characterize anticipatory postural adjustment-reach movement response.

**Results:**

Anodal tDCS over M1 led to significant increase of startReact responses incidence at loud acoustic stimulus time point − 500 ms. Increased trunk involvement during movement execution was found after anodal M1 stimulation compared to PMAs stimulation.

**Conclusions:**

The findings provide novel evidence that impairments in movement planning and preparation as measured by startReact responses for a standing reaching task can be mitigated in individuals with stroke by the application of anodal tDCS over lesioned M1 but not cathodal tDCS over PMAs. This is the first study to show that stroke-related deficits in movement planning and preparation can be improved by application of anodal tDCS over lesioned M1.

*Trial registration* ClinicalTrial.gov, NCT04308629, Registered 16 March 2020—Retrospectively registered, https://www.clinicaltrials.gov/ct2/show/NCT04308629

## Introduction

StartReact (SR) responses triggered by a loud acoustic stimulus (LAS) during the planning and preparation of goal intended actions have been used to probe the state of brainstem neuronal excitability related to posture and movement sequencing [1, 2]. Abnormal posture and movement planning and preparation as shown by an absence of SR responses during standing reaching have been found in previous studies [3, 4]. Premotor areas (PMAs) such as supplementary motor areas and premotor cortex are thought to be involved in posture and movement planning [5, 6]. In preparation for a movement, the neural pathways originating from PMAs to the spinal cord via the reticular formation modulate spinal circuitry through inhibitory effects in order to prevent premature release of the movement [7]. Previous studies also suggested that damage to the premotor cortex following stroke [6, 8] or temporary inhibition by transcranial magnetic stimulation (TMS) over the supplementary motor areas of healthy subjects [7] impair the anticipatory postural adjustments (APAs) preparation during voluntary stepping. Furthermore, animal studies [9, 10] have shown the activation in neurons in subcortical pontomedullary reticular formation (PMRF) were related to the APAs prior to the reaching movement. The signals for the APAs were possibly generated from higher cortical level such as PMAs via cortico-reticular pathway to the PMRF. We proposed that PMAs normally have a modulatory role in SR responses through inhibitory input to brainstem motor circuits and/or spinal cord via the PMRF. Hence, abnormal hyperexcitability in PMAs due to chronic stroke [11] may lead to excessive inhibition of the PMRF and/or spinal cord resulting in an absence of and/or reduced magnitude of SR responses and a disruption of the normal sequencing between posture and movement [4].

Cortical excitability can be modulated by the application of weak continuous direct electrical current over a specific location of the head by noninvasive transcranial direct current stimulation (tDCS) [8]. Depending on the direct current polarity, tDCS can either up-regulate neuronal excitability using anodal tDCS or down-regulate it using cathodal tDCS by hyperpolarizing or depolarizing the membrane potentials [8, 12]. Many studies have demonstrated beneficial effects of applying tDCS over M1 on arm, hand, and lower limb motor performance in individuals with stroke [13–18]. However, only one recent study demonstrated that SR response in ankle dorsiflexion, wrist flexion, and automatic postural responses could be facilitated by applying anodal tDCS over M1 in healthy subjects [19]. No studies have used tDCS over PMAs as a target for neuromodulation therapy to augment posture and movement planning, preparation, and execution following stroke.

The purpose of this study was to determine the modulatory role of the PMAs on SR responses following cathodal tDCS over PMAs in persons with stroke. Knowing that PMAs have projections to the M1, we included anodal tDCS over M1 as a control condition to validate that PMAs stimulation has additional modulatory effects. Our hypothesis was that applying cathodal tDCS over PMAs will reduce the neuronal excitability in PMAs thereby helping to improve posture and movement planning and preparation in persons with stroke, as measured by increased incidence and faster onset of the SR responses.

## Material and methods

### Subject

**Fig. 1:**
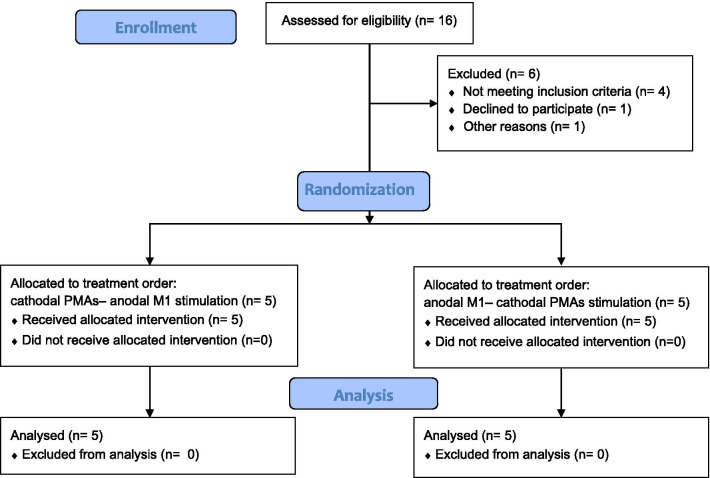
The CONSORT flow diagram showed the recruitment process

Participants were recruited through convenience sampling. The recruitment process flowchart is displayed in Fig. 1. Data were collected from 10 individuals with stroke (Table 1). Inclusion criteria were unilateral cortical or white matter subcortical stroke, age 40 years and older, ≥ 6 months post ischemic stroke or ≥ 12 months post hemorrhagic stroke, residual arm hemiparesis as indicated by Fugl-Meyer Upper Extremity [20] score between 20 and 65, and having the ability to perform reaching movements with the paretic arm in standing without an assistive device. Exclusion criteria included stroke involving bilateral hemisphere, brainstem or cerebellum, any medical condition precluding participation in testing, and other health conditions affecting balance and upper extremity movement function beyond the effects of stroke. Participants were also excluded if they did not meet the TMS safety criterion including having implantable medical devices, history of seizures, taking medications to reduce anxiety, sedatives, and seizure, and pregnancy. All participants gave written informed consent to participate, and the study was approved by the Institutional Review Board at the University of Maryland Baltimore (HP-00064894). Participants were recruited from October 2016 and the data collection of all participants was completed by Oct 2017. There was no deviation from the study protocol. The study was retrospectively registered on ClinicalTrial.gov due to the lack of knowledge about trial registration before enrolment of participants. We confirmed that all ongoing and related trials for this intervention are registered.

**Table 1:** Demographic characteristic

Subject id	Age, years	Sex	Time Poststroke, y	Lesion location	Side of paresis	Dominant side	FM-UE (/66)
#1	75.73	M	14.00	Cortical and subcortical	R	R	49
#2	63.36	M	5.91	Cortical and subcortical	R	R	39
#3	77.63	M	20.51	Cortical	L	L	33
#4	62.58	F	7.55	Cortical and subcortical	L	R	30
#5	68.14	M	8.81	Cortical and subcortical	L	R	62
#6	70.33	F	16.40	Subcortical	R	R	26
#7	74.23	F	51.26	Subcortical	L	L	36
#8	64.10	M	0.97	Subcortical	R	R	65
#9	55.99	M	2.26	Subcortical	R	R	65
#10	79.29	M	1.29	Subcortical	L	R	55
Mean (SD)	69.13 (7.61)	7M/3F	12.00 (15.00)	1 Cortical/5 Subcortical/4 Cortical and subcortical	5R/5L	8R/2L	46.00 (15.06)

*FM-UE* Fugl-Meyer Upper Extremity Score

### Transcranial magnetic stimulation

Motor hotspots were located by using single-pulse TMS delivered by a Magstim 200 stimulator (Magstim Company, Dyfed, UK) using a figure-of-eight coil (70-mm) for biceps brachii and a double cone coil (110-mm) for tibialis anterior. For subjects who had absent motor evoked potentials (MEP) of the affected side, the mirrored location of the nonaffected side hotspot was used to determine the hotspot for the affected side. The active motor threshold was determined while the subjects exerted a force of 20% maximum voluntary isometric contraction (MVIC) of each muscle [25]. The active motor threshold was defined as the lowest stimulus intensity that could evoke a MEP (about 200 μV) in 5 out of 10 consecutive trials during isometric contraction of the tested muscle. A hand-held dynamometer (Chatillon DFX-200 Digital Force Gauge, Itin Scale Co., Inc., Brooklyn, NY) was used to measure the MVIC. The MVIC is defined as the average of 3 measurements of each tested muscle. For each MEP measurement, the assessor holding the dynamometer visually verified if the force reached the level of 20% MVIC and instructed the subjects to hold the level of force exertion. Changes in cortical excitability as measured by MEPs were measured 10 times at the hotspots of the biceps brachii and tibialis anterior with an intensity of the 120% of active motor threshold at 20% of MVIC. A neuronavigation system (Brainsight Version 2, Rogue Research Inc., Montreal, Canada) was used to confirm that the same hotspots were used.

### Transcranial direct current stimulation

**Fig. 2:**
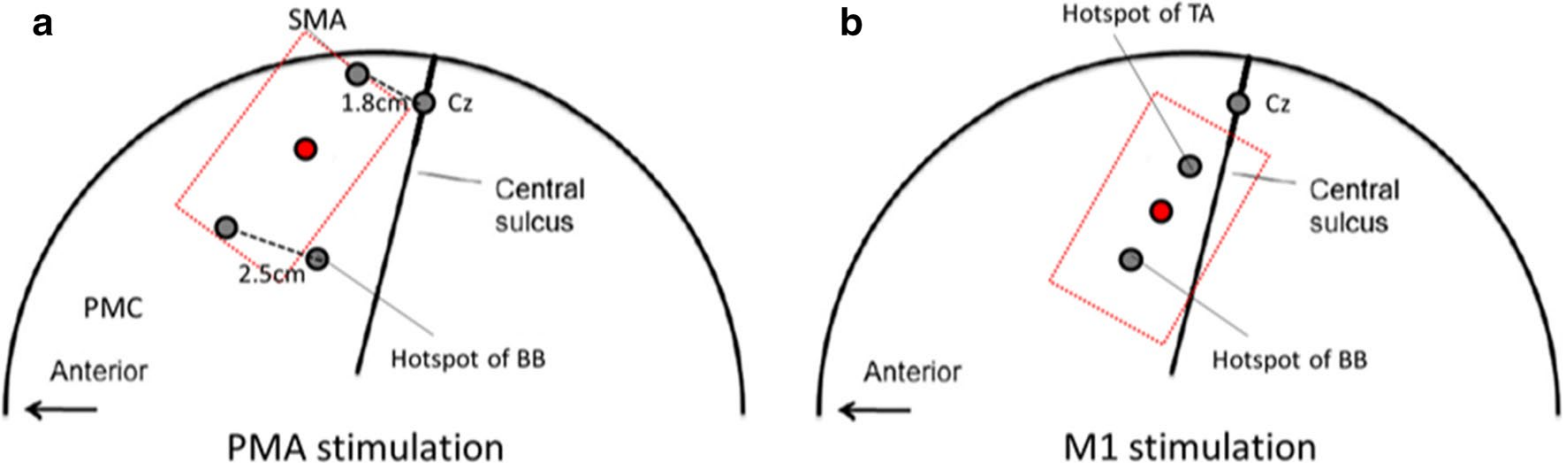
Illustration of the stimulation electrode placements for **a** PMAs stimulation and **b** M1 stimulation during tDCS. SMA, supplementary motor area; PMC, premotor cortex. Supplementary motor area was defined as 1.8 cm anterior to the measured location of Cz [21]. Premotor cortex was defined as 2.5 cm anterior to the motor hot spot of the BB [22]. The hotspot of the BB is normally situated approximately 3 cm lateral and 2 cm in front of the Cz [23] and the hotspot of the TA is situated approximate 2 cm lateral and 1 cm in front of the Cz [24]

TDCS was applied by an iontophoresor (Chattanooga Ionto, Salt Lake City, Utah). The stimulating electrode was placed at the midpoint of the supplementary motor area and premotor cortex for PMAs stimulation (Fig. 2a). Supplementary motor area was defined as 1.8 cm anterior to the measured location of Cz [21]. Premotor cortex was defined as 2.5 cm anterior to the motor hotspot of the biceps brachii [22]. For M1 stimulation, the stimulating electrode was placed at the midpoint of upper and lower extremity M1 where TMS elicits twitches in the biceps brachii and tibialis anterior of the limb respectively (Fig. 2b). The reference electrode was placed on the forehead above the contralateral orbit. Custom-made tDCS electrodes of 15 cm^2^ (3 cm × 5 cm), made of carbon-microfiber material, were thoroughly hydrated by saline (0.9% NaCl) and secured over the subject’s head. One-session of tDCS was administered at an amplitude of 1 mA for 20 min while the subjects were sitting on a chair.

### Instructed-delayed paradigm

**Fig. 3:**
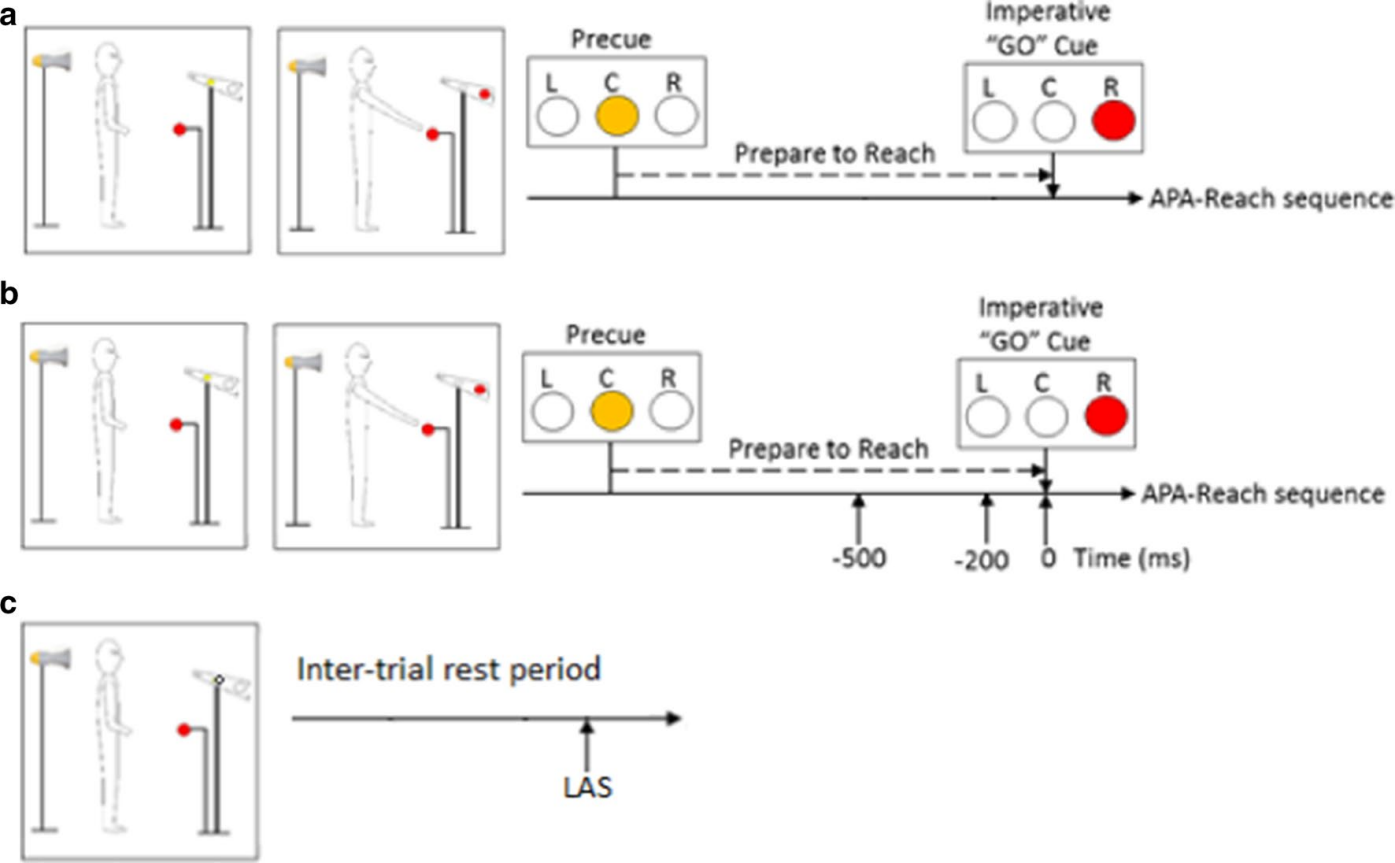
Examples of **a** a right arm control reach trial, **b** a right arm LAS reach trial, and **c** a control LAS trial

A visually cued delayed-response paradigm was used to examine the transition from a stationary standing posture to the rapid initiation of reaching (Fig. 3). Task instruction stimuli were presented using LED lights placed at eye level 3 m in front of the subject. A precue (center) light was presented followed by the imperative "go" cue light with an inter-stimulus delay of 2.5 s. The target ball was placed at 65% of subject’s height and 10 cm beyond subjects’ maximal reach distance of the paretic arms. Subjects were instructed to reach with their paretic arms "as quickly as possible" in response to the "go" cue. An LAS (123 dB, 1 kHz, 40 ms) delivered by a horn speaker (HS-17 T; MG Electronics) placed 30 cm behind subject’s head.

In each testing, subjects performed 65 trials including three conditions: control reach trials, LAS reach trials, and control LAS trials. The order of presentation of LAS (i.e., LAS reach trials and control LAS trials) and control reach trials was partly randomized with the exception that the LAS was not presented during the first five trials and no more than two trials with LAS were presented in a row. Condition 1, control reach trials (45 trials): these trials consisted of standing reach movements performed with no LAS presented. Condition 2, LAS reach trials (3 time points × 5 trials, 15 trials): these trials consisted of standing reach movement performed with the LAS presented at one of the three time points: − 500, − 200, or 0 ms relative to the "go" cue. These time-points were selected based on past normative studies showing progressive increases in the incidence and magnitude of SR responses during this time window reflecting motor preparation [26]. In addition, Condition 3, control LAS trials (5 trials): these trials were collected in which an LAS was delivered during inter-trial standing rest period without reach and without the presence of the precue and go cue, serving as catch trials to verify that in the absence of movement plan, an LAS did not elicit SR response. The number of trials with LAS were kept at 33% of all trials to avoid habituation [27, 28].

### Experimental design

**Fig. 4:**
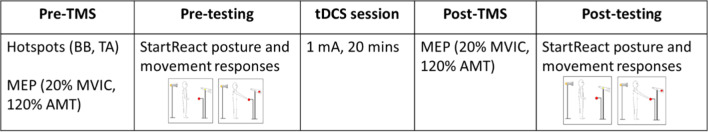
Flowchart of the experimental procedure for each visit. TMS, transcranial magnetic stimulation; tDCS, transcranial direction current stimulation; BB, biceps brachii; TA, tibialis anterior; MEP, motor-evoked potential; MVIC, maximum voluntary isometric contraction; AMT, active motor threshold

Each subject performed two sessions of tDCS over the lesioned hemisphere on two different days separated by at least a 48-h interval: cathodal tDCS over PMAs and anodal tDCS over M1. Knowing that PMAs have projections to the M1, the M1 condition was used to validate that PMAs stimulation has additional modulatory effects. The order of PMAs and M1 stimulation was randomized. Each day consists of a pre-tDCS test, one tDCS session, and a post-tDCS test (Fig. 4). The pre-tDCS testing included finding the hotspots and MEP measurements followed by the standing reaching trials to examine the SR responses. The post-tDCS testing was a repeat of the pre-tDCS testing.

### Data acquisition

Kinetic data including ground reaction forces and moment were collected from two force platforms (AMTI, Watertown, MA) placed beneath the right and left feet at a collection frequency of 600 Hz. Kinematic data were collected at 120 Hz, using a 10-camera Vicon motion analysis system (VICON, Los Angeles, CA). These data were filtered with a low pass, 4^th^ order Butterworth digital filter with a cutoff frequency at 10 Hz [29]. Reflective markers were placed bilateral on subject’s body (see our previous study [4] for detailed placement). Kinematic computations of joint centers were performed using a model [30] written in commercially available software (BodyBuilder, Vicon, Centennial, CO). The muscle activity was recorded from anterior deltoid and biceps brachii of the reaching arm muscle and bilateral tibialis anterior, with a wireless EMG system TeleMyo™ Direct Transmission System (NORAXON, Scottsdale, AZ) using bipolar Ag–AgCl surface electrodes. All electrodes placements followed the recommendations of SENIAM (https://www.seniam.org) [31]. Raw EMG signals were sampled at 1500 Hz. The data for the standing reaching task was bandpass filtered between 30–500 Hz with a 5th order Butterworth filter with Matlab program filtfilt, full-wave rectified, and low-pass filtered (10 Hz Butterworth 4th order) for smoothing purposes. Custom-written Matlab programs (The MathWorks, Inc., Natick, MA) were used to process kinetic, kinematic, and EMG data and all data were verified by visual inspection.

### Data analysis

#### Incidence of StartReact response following LAS

**Fig. 5:**
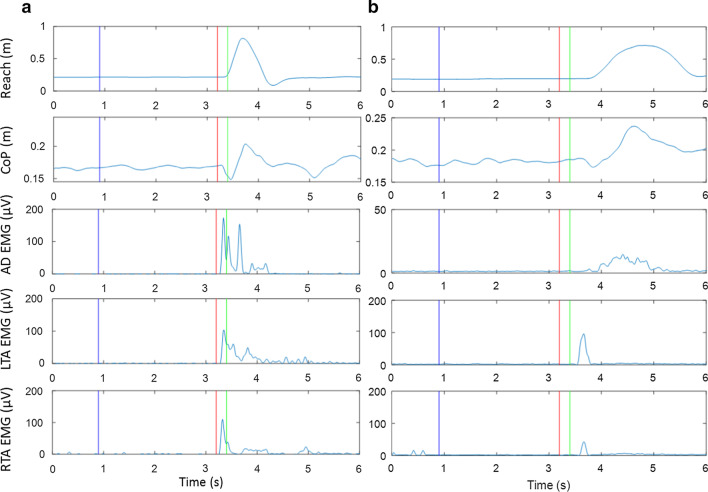
Example of **a** SR response with a completed APA-reach sequence as shown by anterior displacement of hand (top window), posterior center of pressure (CoP) displacement (2nd window), and corresponding muscle activation (3rd–5th windows) after the LAS before the go cue and **b** absent response with neither SR response in APA nor SR response in reach. Blue vertical lines represent the timing of the warning cue. Red vertical lines represent the timing of the LAS. Green lines represent the timing of the go cue. Note that the LAS is at − 200 ms relative to the go cue

Movement planning and preparation were examined using the presence of SR responses (Fig. 5). The incidence of SR responses when the LAS was applied at − 500 ms and − 200 ms was reported. The SR responses for the trials when the LAS was presented at the “go” cue were not determined since the responses evoked by the LAS were possibly intermingled with the responses to the imperative “go” signal. To be considered a SR response in the APA or reach, the occurrence of components of APA or reach were required to be met within one of the following time windows: between the LAS and the go cue, or an early onset of < 3 SDs from the average onset in the control reach condition. The components for an APA response are an initial posterior shift in the center of pressure and an early EMG burst in tibialis anterior before the onset of reach. The components for a reach response are an anterior movement of hand and an EMG burst in anterior deltoid.

#### APA-reach performance

APA and reach onset were defined as the onset of the posterior center of pressure displacement and the onset of the anterior wrist joint center movement with a threshold of 5% peak velocity, respectively. The onset times of muscle activation was calculated based on changes of > 3 SDs for at least 100 ms from the mean signal recorded before the “go” cue or LAS and a continuous increase of muscle activity was seen. The onset times were verified by visual inspection [32].

#### Trunk contribution during movement execution

Trunk flexion was determined by the angular displacement of the line joining the reaching shoulder and the hip joint center on the same side in the sagittal plane at maximum reach normalized by reach distance. Trunk rotation was determined from the angular displacement of the line connecting both shoulders in the horizontal plane in the direction of the reach at maximum reach normalized by reach distance. Pelvic rotation was determined from the angular displacement of the line connecting both hip joint centers in the horizontal plane in the direction of the reach at maximum reach normalized by reach distance. Trunk-pelvic rotation difference was determined from the difference between trunk and pelvic angular displacement at maximum reach normalized by reach distance.

#### Neurophysiological measurement

MEP amplitude was measured by the peak-to-peak EMG amplitude elicited by the TMS.

### Statistical analysis

A linear mixed-effects model using Stimulation (cathodal PMAs vs. anodal M1) and LAS condition (LAS at − 500 ms, − 200 ms, 0 ms relative to the go, and control reach) as fixed factors, and subjects as a random factor was performed to test the effect of cathodal PMAs vs. anodal M1 stimulation adjusting for LAS timing on pre-post change of outcome variables. The model included the main effects of Stimulation, LAS condition, Stimulation × LAS condition interaction, and a random intercept for subjects. Prior to analysis, proportion variables (e.g., incidence of SR response) were corrected for normality using an arcsine square root transformation. Bonferroni adjusted test was used for all post hoc comparison. All outcome variables except for MEP amplitude were transformed and presented as Post–Pre change values. Difference in pre vs. post MEP amplitude was examined by paired t-tests. All statistical analyses were performed by SPSS v.22 (IBM, Armonk, NY). All statistical tests were made at a significant level of *p* < 0.05. All error bars correspond to standard errors.

## Results

### Incidence of SR response following LAS (Fig. 6)

**Fig. 6:**
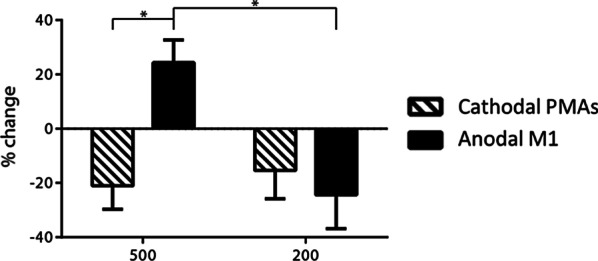
Mean change (± SE) of the incidence of SR response when the LAS was presented at 500 and 200 ms before the go cue

Analysis in the incidence of SR showed differential effects of PMAs vs. M1 stimulation depending on the LAS timing. A significant interaction between Stimulation × LAS condition was found (*F*_(1, 36)_ = 7.246, *p* = 0.011). Stratified analyses showed that SR incidence increased more after anodal M1 stimulation compared to cathodal PMAs stimulation when the LAS was at − 500 ms (*p* = 0.001). In addition, after anodal M1 stimulation, SR incidence increased when the LAS was at − 500 ms but decreased when the LAS was at − 200 ms (*p* = 0.004). In contrast, cathodal PMAs stimulation caused a decrease in the SR incidence at both LAS time points − 500 ms and − 200 ms. This suggests a differential effect of cathodal PMAs vs. anodal M1 stimulation on SR incidence depending on the LAS timing.

### APA-reach performance

**Fig. 7:**
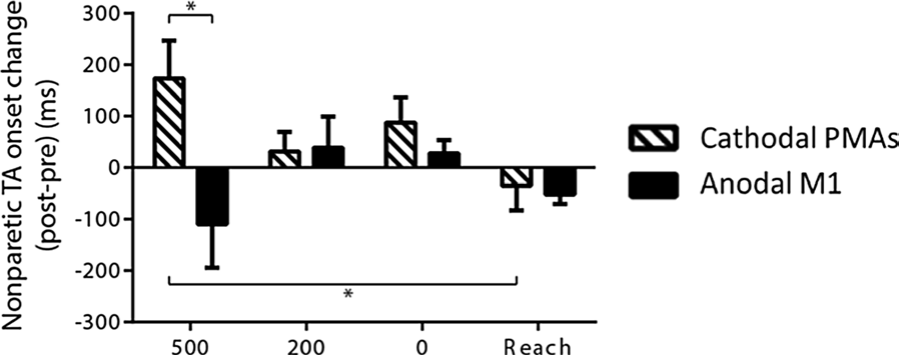
Mean change (± SE) in nonparetic tibialis anterior (TA) onset across conditions (LAS at − 500, − 200, 0 ms relative to the go and the control reach condition). *p < 0.05

There was a significant Stimulation × LAS condition interaction (*F*_(3, 61.587)_ = 3.146, *p* = 0.017) on nonparetic tibialis anterior onset. Stratified analyses showed that there was a significant effect of Stimulation site when the LAS was at − 500 ms (*p* = 0.018). Specifically, the nonparetic tibialis anterior onset was later after cathodal PMAs stimulation than after anodal M1 stimulation at LAS time point − 500 ms (Fig. 7). In addition, after cathodal PMAs stimulation, there was a larger increase of nonparetic tibialis anterior onset time when the LAS was at − 500 ms compared to the control reach condition where there was no LAS applied (*p* = 0.045).

**Fig. 8:**
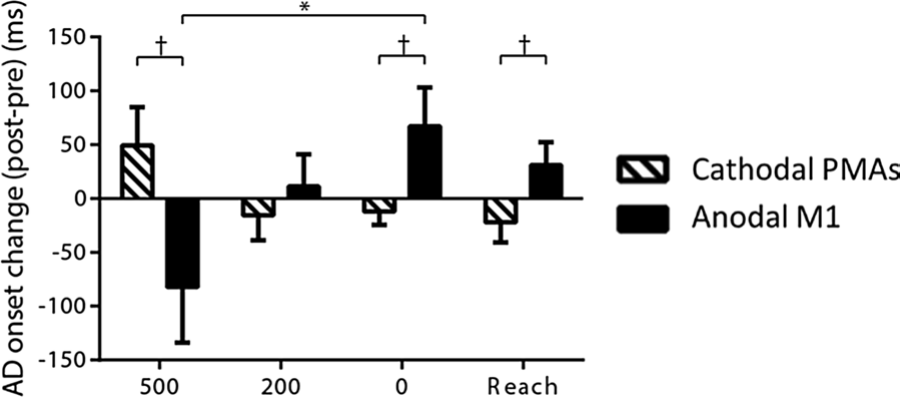
Mean change (± SE) in anterior deltoid (AD) onset across conditions (LAS at − 500, − 200, 0 ms relative to the go and the control reach condition). *p < 0.05 and ^†^*p* < 0.1

There was a significant Stimulation × LAS condition interaction (*F*_(3, 72)_ = 4.708, *p* = 0.005) in anterior deltoid onset (Fig. 8). Stratifies analyses showed that there was effect of LAS condition after anodal M1 stimulation showing that a decrease in anterior deltoid onset when the LAS was at − 500 ms compared to when the LAS was at 0 ms (*p* = 0.026). Stratified analyses also showed that there were effects of Stimulation when the LAS was at − 500 ms (*p* = 0.05), 0 ms (*p* = 0.053), and control reach condition (*p* = 0.058) although the difference was outside the significance cutoff. When the LAS was at − 500 ms, the increase of anterior deltoid onset was larger after cathodal PMAs stimulation than anodal M1 stimulation. However, when the LAS was at 0 ms or in the control reach condition, the increase of anterior deltoid was larger after anodal M1 stimulation than cathodal PMAs stimulation. There were no main effects of Stimulation, LAS condition, and Stimulation × LAS condition interaction on other APA-reach performance variables.

### Trunk contribution during movement execution

**Fig. 9:**
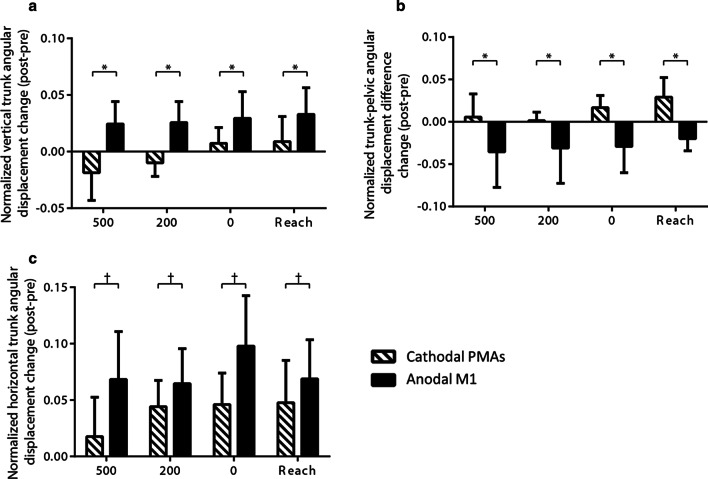
Mean change (± SE) in **a** trunk flexion, **b** trunk-pelvic rotation difference, and **c** trunk rotation across conditions (LAS at − 500, − 200, 0 ms relative to the go and the control reach condition). *p < 0.05 and ^†^ *p*< 0.1

A significant main effect of Stimulation was found on the trunk flexion (*F*_(1, 66)_ = 8.622, *p* = 0.005) and the trunk-pelvic rotation difference (*F*_(1, 75)_ = 4.721, *p* = 0.033). The trunk flexion and the trunk-pelvic rotation difference had a larger increase after anodal M1 stimulation compared to after cathodal PMAs stimulation (Fig. 9a, b). Marginal main effects of Stimulation on trunk rotation was found (trunk rotation: *F*_(1, 66)_ = 3.294, *p* = 0.074). After anodal M1 stimulation, there was a trend of greater increase in the trunk rotation compared to after cathodal PMAs stimulation (Fig. 9c). No significant main effects of LAS condition and Stimulation × LAS condition interaction were found for the above outcome variables.

### Neurophysiological measurement

The number of subjects who we were able to retreive MEPs for biceps brachii was 6 and for tibialis anterior was 8 subjects for the anodal M1 stimulation. For the cathodal PMAs stimulation, we were able to retreive MEPs in 5 subjects for biceps brachii and in 7 subjects for tibialis anterior. No significant difference in MEP amplitudes before and after tDCS was found.

## Discussion

**Fig. 10:**
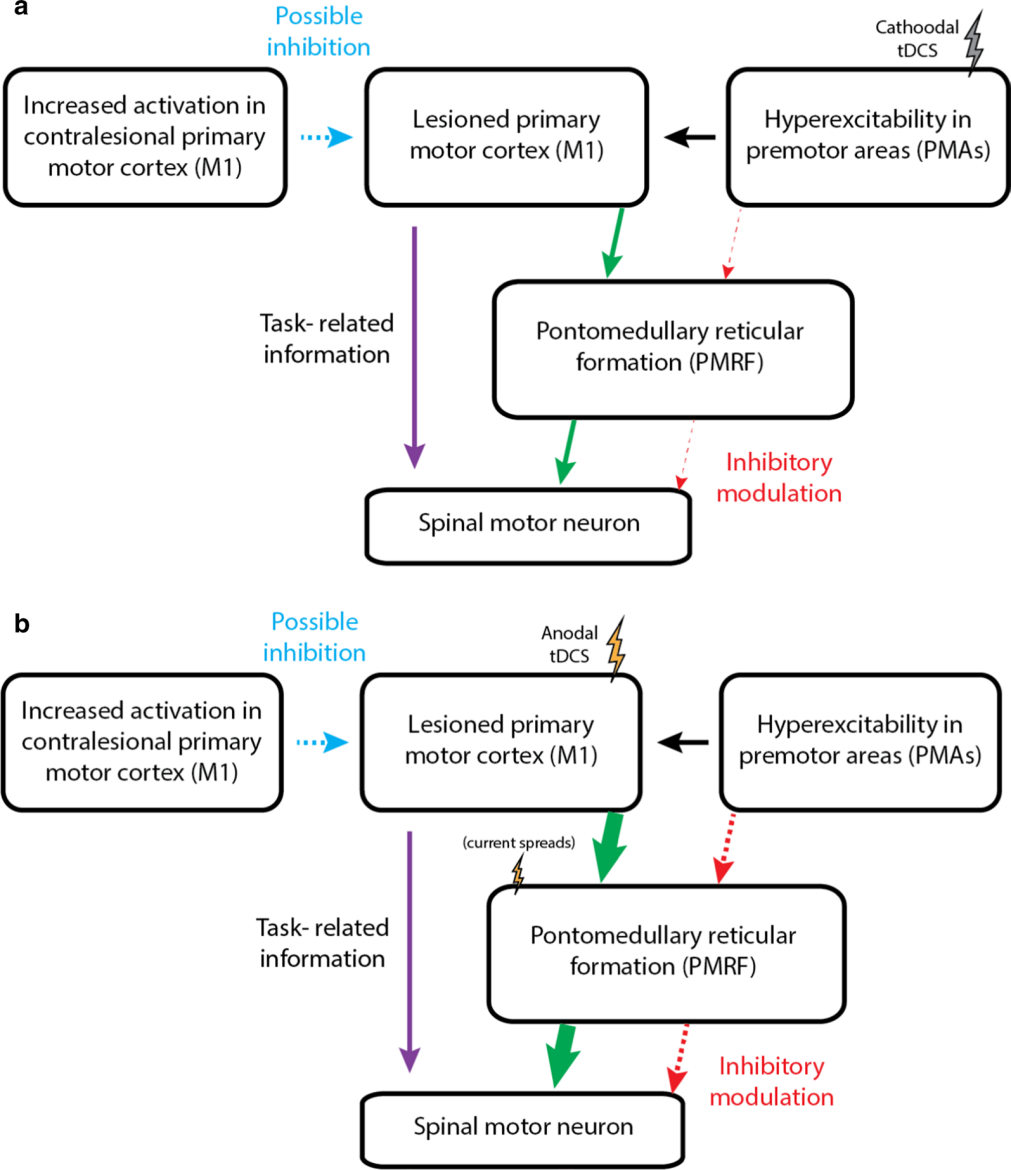
Schematic representation of proposed neuromodulation of **a** cathodal tDCS over PMAs and **b** anodal tDCS over M1 related to posture and movement planning and preparation in individuals with stroke. In **a**, we hypothesized that cathodal tDCS over PMAs would down-regulate the hyperexcitability in this region and consequently restore the excessive inhibitory input (red dashed arrow) from PMAs to the PMRF via cortico-reticulospinal tract. In **b**, based on our findings, it is possible that application of anodal tDCS over M1 facilitates the subcortical PMRF via the cortico-reticular drive (green arrow) or direct excitations caused by the applied current

The findings that anodal tDCS over M1 was more effective in increasing the SR incidence than cathodal tDCS over PMAs in individuals with stroke was unexpected. It has been suggested that PMRF is critically involved in generating posture responses [9, 10] and in the SR responses [19].We proposed that the abnormal hyperexcitability in PMAs due to chronic stroke may lead to excessive inhibitory input via the cortico-reticulospinal pathway, and consequently impairs posture and movement planning, preparation, and execution. Therefore, down-regulating the hyperexcitability in PMAs by applying cathodal tDCS over these regions may restore the inhibitory input from PMAs to the PMRF (Fig. 10a). However, opposite to our hypothesis, our findings showed an increase in SR incidence and a decrease in muscle activation onset latency when the LAS was applied at − 500 ms after anodal M1 stimulation compared to cathodal PMAs stimulation in individuals with stroke (Fig. 10b). This is consistent in part with Nonnekes et al. [19] who showed a decrease in reaction time irrespective of whether or not an LAS was given at the imperative stimulus after anodal M1 stimulation. The author concluded that the subcortical structures can possibly be facilitated by an enhancement of the cortico-reticular drive or by direct excitations caused by the applied current. One animal study also showed that tDCS over the motor cortex of anesthetized cats facilitated subcortical structures either directly or indirectly [33]. Moreover, Wagner and colleagues showed possible direct subcortical facilitation from the spread of current during tDCS application and importantly the current density distributions were different in the stroke model [34]. It is possible that application of anodal tDCS over M1 facilitates directly or indirectly the subcortical structures such as PMRF. With our stimulation paradigm, rather than remediating the excessive inhibitory input from the PMAs onto the subcortical brainstem by applying cathodal tDCS over PMAs, the direct or indirect subcortical facilitation from anodal tDCS over the region of M1 may be more effective modulating brainstem neuronal excitability and in turn improving movement planning and preparation. Another plausible explanation is that given that PMAs include a larger cortical area compared to M1, a higher intensity or longer duration of tDCS is required to induce changes in PMAs. Although the findings from this study are not consistent with our hypothesis, our conceptual model does not mutually exclude the possible subcortical facilitation mechanisms.

Another factor that influenced the effect of tDCS on the incidence of SR response was the LAS timing. A greater increase in the incidence of SR responses following anodal M1 stimulation was only found at the LAS time point − 500 ms but not at − 200 ms. One possible explanation is that during the time course of movement planning and preparation, the excitability at the cortical and subcortical levels changes gradually. During preparation for movement there are two pathways controlling spinal motorneuronal circuitry in order to preplan the movement and avoid premature release of the movement [7]. The excitatory input which originates from PMAs and relays via M1 to spinal cord transmits task-related information while the global inhibition originates from PMAs and relays via subcortical level to spinal circuity (i.e., cortico-reticulospinal pathway) prevents premature release of a motor action. It has been suggested that the corticospinal excitability during movement planning and preparation undergoes progressive changes due to global inhibition [35]. The dynamic changes of inhibitory inputs onto spinal motorneuronal circuitry during movement planning and preparation may lead to differential effects of tDCS on the incidence of SR responses depending on the LAS timing.

There were no detectable neurophysiological change of MEP amplitudes following PMAs and M1 stimulation but posture and movement planning, preparation, and execution were modified after tDCS. One possible reason for non-significant changes in MEP amplitudes is inter-individuals response variability. Previous studies have reported that the effects of tDCS on MEP amplitudes elicited by single-pulse TMS as a measure for corticospinal excitability are highly variable [36, 37]. Another possible reason is that the changes in cortical excitability after tDCS are the result of intracortical facilitation or inhibition not corticospinal excitability as shown in MEP amplitudes elicited by single-pulse TMS. One study by Nitsche et al. [38] used paired-pulse TMS with different interstimulus interval and found that following tDCS intracortical inhibition and facilitation were modified. This may suggest that single-pulsed MEP amplitude may not be a preferred indicator of neurophysiological changes obtained by tDCS.

Our findings show a differential effect of cathodal PMAs vs anodal M1 stimulation on trunk contribution during reaching execution across LAS conditions. Generally, we found that there was a greater increase in trunk movement after anodal M1 compared to cathodal PMAs stimulation. One possible explanation is that the anodal electrode placement over M1 stimulation included the trunk representation since it was at the midpoint of hotspots of biceps brachii and tibialis anterior. Based on the homunculus map of a human brain, the area representing the trunk is in the middle of arm and leg areas. Thus, the anodal M1 stimulation may also affect trunk performance. Another plausible explanation is the subcortical facilitation described in the previous section. Since the PMRF is known to be involved in generating compensatory postural responses [9, 10], the direct or indirect facilitation induced by anodal M1 stimulation may increase the excitability in the PMRF and subsequently alter trunk involvement during reaching.

One major challenge of our study was that we aimed to modulate PMAs and M1 separately. Even though small tDCS electrodes (15 cm^2^) were used over both target areas in an attempt to increase the focality of stimulation, the possibility that during PMAs stimulation, M1 region was also partly stimulated can not be ruled out, and vice versa. Nevertheless, differential effects of cathodal PMAs vs. anodal M1 stimulation on variables of posture, movement planning, preparation and execution were demonstrated in the present study and provide credible evidence that modulation of these two areas by tDCS is plausible. Another limitation is that the absence of detectable MEP amplitudes changes following tDCS. The difficulty to record MEPs data in 20–30% of our stroke subjects, inter-individuals response variability, and inability to capture changes in cortical excitability by single-pulsed TMS protocol possibly contributed to nonsignificant changes in MEP amplitudes. Future studies should also consider measuring cortical excitability changes following tDCS with a more comprehensive assessment by TMS such as cortical silent period in order to detect the modulation effect of tDCS.

## Conclusions

The present results show that following the application of cathodal and anodal tDCS over the region of the PMAs or M1, respectively, “stimulation”-specific changes were observed in posture and movement planning, preparation and execution in individuals with stroke. We also provide novel evidence that stroke-related deficits in movement planning and preparation as shown by an abnormal absence of SR responses can be improved by application of anodal tDCS over lesioned M1 and the enhancement effects are depending on the timing of the LAS. It is possible that either direct or indirect subcortical facilitation resulting from the anodal tDCS over M1 may offer a new neuromodulatory target to remediate the imbalance in neuronal excitability between PMAs and subcortical brainstem level, which in turn improve the posture and movement planning, preparation, and execution in individuals with stroke.

## Data Availability

The datasets used and/or analysed during the current study are available from the corresponding author on reasonable request.

## Abbreviations

BB: Biceps brachii
TA: Tibialis anterior
MVIC: Maximum voluntary isometric contraction
AMT: Active motor threshold
SMA: Supplementary motor area
PMC: Premotor cortex
APA: Anticipatory postural adjustment
LAS: Loud acoustic stimulus
M1: Primary motor area
MEP: Motor evoked potentials
PMAs: Premotor areas
PMRF: Pontomedullary reticular formation
SR: StartReact
tDCS: Transcranial direct current stimulation
TMS: Transcranial magnetic stimulation
